# Contentious Issues and Future Directions in Adolescent Gambling Research

**DOI:** 10.3390/ijerph182111482

**Published:** 2021-10-31

**Authors:** Paul Delfabbro, Daniel L. King

**Affiliations:** 1 School of Psychology, The University of Adelaide, Adelaide, SA 5005, Australia; 2 College of Education, Psychology and Social Work, Flinders University, Bedford Park, SA 5042, Australia; Daniel.King@flinders.edu.au

**Keywords:** adolescent gambling, development, methodology, problem gambling

## Abstract

Background. There is currently considerable public policy and regulatory interest in the nature and prevalence of underage gambling. Research in this area has purported to show that adolescents are at elevated risk of problem gambling and that early exposure to gambling or gambling-like activities could be a potential precursor to future harm. Method. In this commentary, we provide a critical appraisal of these arguments with reference to major studies in the field of gambling studies. It is argued that adolescent gambling research is a contentious area. Some questions remain concerning the validity of adolescent problem gambling measures, the strength of the association between adolescent and adult gambling and the impact of simulated gambling activities. Results. The paper summarises the conceptual and methodological issues that should be considered and addressed in future studies to strengthen the validity of research in this area. Conclusion. The paper encourages the greater use of harm-based measures, longitudinal and individual-level transition analyses and questions that capture the influence of activities rather than just their temporal sequencing.

## 1. Introduction: Underage Gambling

Although commercial gambling activities are not legally available to young people under the age of 18 years in most countries, there is now around three decades of research to show that underage gambling or gambling-like activity occurs quite regularly. Not only are adolescents able to access adult gambling venues, but the advent of online gambling now enables them to gamble online by bypassing age verifications or by asking people to gamble on their behalf [1,2,3,4]. Young people are also able to gain access to gambling with the help of others, such as when parents or older siblings place wagers on their behalf or buy them lottery tickets as gifts. These various access points and social pathways into gambling activities make adolescent gambling a complex phenomenon to study in research (e.g., distinguishing mere exposure from active participation) as well as a multi-faceted issue for public policy and regulatory discussion [5].

Studies into the nature and prevalence of adolescent gambling have emerged from different parts of the world, highlighting that many adolescents report past-year involvement in gambling activities [6,7,8,9,10]. In Australia, for example, a study by Dowling et al. [11,12] based on 612 students aged 12–18 years sampled from secondary schools in the Australian State of Victoria reported that 67.5% young people had gambled at least once in the previous 12 months, with scratchies (48%), card games (42%), horse-racing (22%) and sports betting (19%) found to be the most popular activities. In Canada, a study by Wijesingha et al. [13] conducted in three provinces found that 41% of adolescents reported having gambled in the previous 12 months: 32% had gambled on land-based activities and 9% online. Similar studies conducted in the UK by the UK Gambling Commission (e.g., in 2018) [10] report similar results. In 2018, a survey of 2865 young people aged 11–16 years reported that 39% reported having gambled with their own money in the previous year with 14% having done so in the last week. Other studies show that the annual prevalence of online gambling in adolescent populations varies by country. Elton-Marshall, Leatherdale and Turner [14] in Canada (in a study of over 10,000) teens produced an estimate of 10%. Another study by Canale, Griffiths, Vieno and Siciliano [15] in Italy had an estimate of 15%, and Olason et al. [16] obtained a figure of 24% in Iceland.

There is also now increasing recognition that young people can legally and easily engage in gambling-like activities in the form of social casino games (e.g., social media games, which include *Slotomania*, are realistic simulations of realistic casino games played usually for points or credits) on smart devices or via other online games that feature realistic gambling environments [17]. Video games also now feature an increasing number of monetised reward structures, including the requirement or strong incentive to purchase additional items or content to progress in the game or to gain status. Some of these features, including loot boxes, can allow gamers to make repeatable purchases where the outcome is determined by chance [18] or optimised by the game’s systems to facilitate continuous spending [19]. Loot boxes (which can include randomised card draws) are a form of online lucky dip which can be earned through gameplay or purchased to yield randomly generated game features. This increasing monetisation of gaming is seen as blurring the boundaries between gambling and gaming [20] and is thought to be a potential influence on the gradual uptake of gambling activities by young people.

As with studies of commercial gambling, there is also consistent evidence that simulated or gambling-like activities are popular among some young people. In 2018, the UK Gambling Commission reported that 13% of 11–16-year-olds had played online gambling-style games and 12% followed gambling companies on social media. In Australia, King et al. [21] surveyed 1287 young people aged 12–18 in high schools in Australia and found that 10% of adolescent respondents had tried gambling apps on Facebook, 26% had played video games with gambling content and 5% had tried demo or practice modes on Internet gambling sites. In both studies, engagement with monetary gambling was found to be associated with simulated gambling. Multiple studies have now been undertaken to confirm that loot boxes are a popular feature of video games and that young people are willing to purchase these features, if they do not win them in games. Zendle, Meyer and Over [22] showed, for example, that in a sample of 1188 gamers aged 16–18 years recruited online, 47% had purchased a loot box and that those who scored higher on the Problem Gambling Severity Index reported greater loot-box expenditure and that they had purchased a loot box. In fact, 84% of problem gamblers detected in the sample had purchased a loot box compared with 25% of non-problem gamblers.

Another important area of adolescent gambling research is work that has attempted to measure the prevalence of problem or pathological gambling. A consistent finding in most of this research is that the rates for problem or pathological gambling often tend to be higher in adolescent studies than in adult studies. As indicated in a systematic review by Calado, Alexandre and Griffiths [6], for example, prevalence figures have ranged from 0.2 to 12% with mean figures usually around 3–4%, which is many times higher than the corresponding adult rate which rarely exceeds 1–2% in most studies [2,23,24]. These elevated figures have been obtained in research conducted in the United Kingdom [10,25,26], the United States [9,27,28,29], Canada [2,3,30] and in Australia [7,31]. These findings are thought to coincide with studies of adult populations that commonly show elevated rates of problem gambling in younger adults (e.g., 18–24 years) which suggest that adolescence may often be the starting point for problems that manifest themselves more strongly in early adulthood [32].

## 2. Summary and Aims of Paper

When all these various findings are combined, it would appear that significant concerns about adolescent gambling are justified amongst policymakers, regulators, educators and parents. The evidence suggests that adolescence is a period during which young people are introduced to gambling, exposed to gambling and where gambling, or gambling-like experiences begin, and that it may be establishing a foundation for future gambling or gambling problems. Despite evidence that adolescent gambling participation rates may be falling [10], researchers point to new emerging influences and threats. These include the emergence of gambling-like activities in video-gaming (described above) as well as the rapid proliferation of sports advertising that is often difficult for young people to avoid online, at sports matches, and when they watch TV [33]. Youth gambling is now often prioritised as an area of policy and research interest and reforms of gambling accessibility, its advertising and promotions and its interaction with sport is a topic of considerable regulatory interest.

On the whole, the body of evidence supporting the engagement of adolescents in gambling appears to be reasonably consistent [34] and it supports the importance of maintaining vigilance as well as targeting adolescence as an important age for early intervention and education. However, as we outline in this paper, the field of adolescent gambling studies has also raised a number of contentious issues and questions that have not been fully addressed in the existing literature. These issues relate to a variety of issues, including the validity of measures used to capture adolescent gambling and problem gambling; the strength of the association between adolescent and adult gambling; whether exposure to gambling or gambling-like content necessarily gives rise to problem gambling and challenges with contacting high-risk populations. In this paper, we discuss these different issues and then conclude with an overview of conceptual and methodological issues that we believe should be addressed in future studies to enhance the validity of adolescent gambling studies.

### 2.1. Issue 1: Elevated Prevalence Rates in Adolescent Samples

The fact that adolescent studies often yield prevalence rates higher than adult populations is often taken to indicate that young people are experiencing more problems with gambling. Such estimates are usually based on the administration of adolescent problem gambling measures such as the DSM-IV-J (either binary or multiple response versions) [24,35], SOGS-RA [3,36] or the Canadian Adolescent Gambling Inventory (CAGI) [37]. These measures are based on the original DSM-IV classification for problem gambling and adapted for adolescents and usually refer to “pathological” or “problem gambling”, although the modern DSM-V classification uses the term “disordered gambling” (the general term will be used in this paper as well as terminology used in the source articles). Items relate to preoccupation (thinking too much about gambling); withdrawal (feeling bad or fed up when they have tried to cut down on gambling); tolerance (needing to gamble more to gain the same excitement) and other items relating to impaired control, chasing and engaging in anti-social behaviour such as spending one’s lunch money on gambling/stealing or telling lies [10]. The multiple-response version of the instrument tries to strengthen measurement by asking about the frequency of gambling and this is thought to avoid an over-reliance on binary items that might refer to infrequent behaviours.

In principle, this approach to measurement appears logical, but questions have been raised about the challenges associated with this approach [38]. The first question is to do with language. As Ladouceur et al. [39] have observed, when adolescents are given similar questions as adults about their gambling, it is not clear that they understand the questions in quite the same way. The authors showed that when more detailed explanations were provided as to the meaning of screening questions, the level of endorsement (and therefore the pathological gambling rate) significantly declined. A second issue is the conceptual similarity or whether the meaning of questions is similar for both adolescents and adults. For example, in adolescent measures, committing illegal acts can include taking lunch money from or using money that should be used for different purposes. In adults, this could be criminal behaviour including embezzlement. Another example is questions relating to relationships. Adolescents might report having an argument with a parent or peer over gambling, but this is unlikely to be a precursor to marital dissolution or family breakdown. Similarly, although adolescents might report spending their pocket or fare money to gamble, this is unlikely to have the same consequences as draining a bank account, losing significant assets or getting involved with payday lenders or loan-sharks. In other words, it is possible for adolescents to endorse many items on these measures and reach the threshold for pathological or problem gambling without necessarily experiencing very significant problems with gambling. In other words, despite responding to items that share similarities with adult measures, it does not follow that adolescent pathological gambling measures capture quite the same construct or to the same severity. Thus, while these measures might have acceptable psychometric properties and appear capable of capturing a valid construct (e.g., higher risk gambling in adolescents), they may not be capturing the same types of behaviours indicative of a gambling disorder as captured by adult measures. For this reason, it may not be valid to compare adolescent figures for pathological gambling with those obtained using adult samples. The greater apparent prevalence of pathological gambling in adolescents may, therefore, not exist, or may need explanation because adult and adolescent “pathological gambling” are two different things.

In this paper we will point to several lines of evidence that raise questions about the prevalence of adolescent pathological gambling. The first of these is the lack of serious evidence of harm. In several studies, questions have been included that ask about the amount of money young people are spending on gambling (e.g., per week or month). These studies show that the level of expenditure is often quite low. For example, in an Australian study by Lambos et al. [40] involving over 2700 adolescent gamblers, the mean weekly expenditure was AUD 10 or less. Similarly, in the 2018 Gambling Commission (2018) report in the UK [10] which surveyed 11–16-year-olds about their gambling habits, it was found that the previous week’s expenditure reported by young problem gamblers was only GBP 29, which is not much more than the cost of 1–1.5 daily coffees. The 2018 UK Gambling Commission report also reported some of the lowest adolescent weekly participation rates obtained since the survey had first been commissioned in the mid-2000s, and yet, the prevalence rate for pathological gambling was 1.7% (over twice the UK adult rate of 0.7% obtained in 2012). A figure of 1.7% is considerably higher than all adult rates obtained in Australia over the last 20 years and in a country with the highest per capita expenditure on gambling in the world [32]. 

### 2.2. Issue 2: Limited Evidence of Harm

Most inferences about the harm associated with adolescents have arisen from correlational evidence. In general, what this work shows is that higher risk adolescent gambling is associated with poorer outcomes on a range of other measures. Adolescents with gambling problems have been found to have higher rates of petty criminal behaviour, substance abuse and truancy [3,24,25,41,42]. Adolescent gambling has also been associated with risky driving and underage drinking [41,43]. Furthermore, Delfabbro, Grabosky and Lahn [44] reported that among adolescent problem gamblers in the ACT, smoking rates were four times higher, marijuana use was six times higher and hard drug use was 20 times higher than in their non-problem gambling counterparts. There are also links with poorer developmental outcomes, including reduced educational performance [3,24,25,26,45,46]; satisfaction with school [43], reduced engagement with school [47,48]; and disrupted study related to their need to gamble [7]. Psychological adjustment scores tend to be poorer in higher risk adolescent gamblers [49]. 

Although such evidence provides support for the notion that adolescent pathological or problem gamblers are generally a higher risk group, it does not necessarily show that gambling is the cause of the problems observed. Instead, the effects observed could be strongly influenced by selection effects. Young people who have other underlying social or psychological issues, an interest in risky activities and risk-taking tendencies, or who are generally more impulsive may be attracted to gambling because: (a) it provides a source of stimulation or excitement and/or (b) an escape from other problems [41,50]. Thus far relatively few attempts appear to have been made to capture the harms directly associated with adolescent gambling as opposed to other broader factors [51]. Although some evidence of truancy and petty crime is evident in some studies [42,52], evidence in support of significant financial harm associated with adolescent gambling is less common.

### 2.3. Issue 3: Low Help-Seeking Rates

Perhaps one of the strongest sources of doubt about adolescent prevalence figures is that very few adolescent problem gamblers are referred to or present to treatment services [53,54,55]. This has amplified concerns that the measurement tools used to assess youth gambling may yield over-estimates. However, in defence of adolescent gambling research, Gupta and Derevensky [23] report that many adolescents do not present themselves to treatment because of embarrassment or because they do not feel that they need help. For parents who take their adolescents to a mental health service, gambling may not be a concern and may not have been detected; instead they may be seeking assistance with anger or aggressive behaviours or school-related concerns. Young problem gamblers may also have others who can bail them out. Funded services for problem gambling may also not be designed for this population or be appropriate for them. Gupta and Derevensky argue that they have had clinical experience with adolescent problem gamblers in the age of 14–21 years, but it is not clear how many of these were under the age of 18 years. Moreover, they point out that few validated treatments for adolescent problem gambling have been developed because an adequate population of clinical cases of adolescent problem gambling has been difficult to establish.

These views are borne out in studies of treatment providers by Rigbye [55] as well as the review by Chevalier and Griffiths [53]. At the individual level, adolescents might mature out of the problem; be less likely to recognise the problem; might experience only short-term harm or receive “bail-outs” from others. At the service level, Rigbye points out that there can be clinical barriers (e.g., services lack the skills to deal with youth problems); environmental barriers (e.g., a lack of youth-specific services); motivational barriers (e.g., adolescents do not feel the need to seek help); and client-level barriers (e.g., adolescents are not aware of services, able to engage with them, or do not believe that formal help-seeking services are the best option for them [23]. All of these points appear to be valid reasons why adolescents do not seek help, but evidence sourced from young adolescent problem gamblers is rarely reported so that it remains unclear whether the lack of help-seeking is due to these inferred barriers as opposed to an over-estimation of the problem.

### 2.4. Issue 4: Precursor to Adult Gambling: Longitudinal Evidence

Another important area of research that is needed to validate youth gambling research is to show a connection between adolescent gambling and adult gambling [56]. In other words, is youth gambling and problem gambling a predictor of gambling or gambling-related problems? At present the findings appear to vary depending upon the methodology used. Evidence in support of stability in gambling habits arisen from studies involving successive cross-sectional designs [31,57,58]. These studies show some evidence of consistency in the percentage of people reporting gambling or gambling-related problems. However, these studies often compare different samples rather than the same people. For this reason, it is important to examine studies that have tracked the same cohort of individuals over time. Winters, Stinchfield, and Fulkerson [28], for example, tracked 532 young people (originally aged 15–18 years) from a previous telephone survey in Minnesota and showed that overall gambling participation rates, as well as rates for particular activities, remained very stable from one year to the next. Similarly, Vitaro et al. [50] showed that young Canadian adolescents (age 12–13 years) with higher impulsivity scores and who gambled at this early age were significantly more likely to report problems with gambling at the age of 17 years. Slutske, Jackson and Sher [59] examined the stability of gambling patterns in a cohort tracked from the age of 18 to 29 years, and also in adolescent research led by Winters [60,61]. These studies showed evidence for stability over time, with the proportion of young people displaying problematic levels of gambling remaining very stable from adolescence to adulthood.

Unfortunately, as Winters et al. [62] have pointed out, such studies often only involve comparisons of data at a group level [60,61,63]; they do not indicate the stability of individual behaviour. Some young people may have stopped gambling altogether, while a similar number may have commenced gambling, but such changes would have been masked by the overall figures. Evidence, in fact, suggests that problem gambling is often transitory or episodic [59,62]. Those who report being problem gamblers at one point in time often report having no difficulties when interviewed at another point. For example, Winters et al. [62] conducted a study involving that tracked 305 young people from mid-adolescence and showed that only 29% of problem gamblers at time one were still problem gamblers by early adulthood (aged over 17 years), although early problem gambling was still moderately associated with later problem gambling. Similar findings emerge in other studies [34,64,65,66]. In an Australian study, Delfabbro, Winefield and Anderson [34] examined the gambling habits of 578 adolescents aged 15–16 years that were tracked over four years (until all were adults aged 18–19 years). The results showed that gambling habits are very unstable over time. For instance, young people who gambled in one year on a particular activity did not necessarily gamble on that same activity in other years. Only around 10% of the sample reported gambling both during adolescence and adulthood on individual activities. Participation in individual activities at the age of 15–16 years generally did not predict involvement at 18–19 years, but stronger associations were obtained for gambling at 16–17 years and adult gambling.

Similarly, Delfabbro, King and Griffiths [34] tracked 256 young people from age 16 to age 20 with interviews conducted every year. It was found that young people’s gambling habits varied considerably from one year to the next. Only a relatively small proportion of the sample reported gambling on any one type of gambling in all four surveys. There were also few significant associations between gambling participation at age 16–17 years and participation four years later. Little consistency was found in respondents’ reporting of problems related to gambling. These Australian findings are generally supported by British research reported by Hollen et al. [65] involving a three-year longitudinal study. They reported that although there was some consistency in gambling in general, only 43 out of 1692 young people tracked from age 17 to 24 years consistently reported being regular gamblers. Other studies [66] suggest that the adolescent–adult gambling association is probably best studied using some form of trajectory analysis based on the analysis of groups of young people who follow different pathways from adolescence in adulthood. What this research shows is that there appears to be a group of higher risk individuals who are more likely to remain high risk over time; that is, their early gambling tends to predict their subsequent gambling [63] However, this once again appears to support the view that gambling during adolescence per se (for the vast majority of young people) is usually not a precursor of ongoing problems or even a maintenance of gambling habits.

### 2.5. Issue 5: The Gateway Hypothesis and Activity Transitions

Another important issue in adolescent gambling research is the influence of gambling-like activities on subsequent adult gambling. To what extent does exposure to simulated gambling activities, video games and features such as paid loot boxes influence gambling? [17,21,67,68,69,70,71]. Internationally, there has been considerable policy and regulatory concern about whether exposure to gambling-like content might prime young people to gamble or “normalise” gambling and lead to potential future harm [18,72,73]. Studies that have examined the overlap between social casino games and gambling show that young people who are more likely to gamble are also more likely to play social casino games. Higher risk or problem gamblers are also more likely to purchase loot boxes when they play video games [74,75]. These findings have, therefore, led to arguments that these activities might be a “gateway” to gambling.

A review of this evidence, however, shows that many of the findings are correlational and do not necessarily support a causal relationship between the two classes of activity. For example, in studies that have examined whether young people have migrated from simulated to regular commercial gambling, only a minority of young report having made this transition. For example, Kim, Wohl, Salmon, Gupta, and Derevensky [76] examined the adult migration from social casino games to financial gambling by surveying 409 social casino gamers recruited from Amazon’s Mechanical Turk system who had never gambled previously. Over a six-month period, the researchers examined whether young people had transitioned to gambling. The results showed that 26% had done so. In another study in Australia, Gainsbury et al. [69] reported that the majority of respondents (71.2%) did not believe that social casino games had affected their gambling. Only 9.6% reported that their gambling overall had increased and 19.4% reported that they had gambled for money as a direct result of these games. In other words, young people are more likely to report that exposure to these games made little difference.

In a review of the loot-box literature, Delfabbro and King [77] argue that the gateway hypothesis has to be applied with considerable caution because associations between problem gambling and loot box use could be a selection effect and have little to do with exposure to these video-game features. Instead, they argue that those young people who have a stronger interest in gambling are more likely to gravitate towards gambling-like content in video games because this is consistent with their activity preferences. In other words, people who are already gamblers will play loot boxes rather this activity turning people into gamblers. Moreover, they argue that caution must be applied whenever attempts are made to draw inferences from what is observed in adolescence in adult studies. Just because one behaviour (namely, gambling) appears to follow after another activity (social casino games, video games or loot boxes) does not mean that the former is caused by the latter. This sequencing of activities only occurs because the simulated and video-game activities are legal for under young people under 18, whereas commercial gambling is not. Thus, it is not surprising to find that video games or simulated gambling precede commercial gambling. To argue that the two are causally related, without further evidence, is rather like arguing that drinking soft drinks before the age of 18 is a gateway product to drinking alcohol at age 21 years. For researchers to show a causal connection, they need to ask more specific questions relating to the influence of simulated gambling and examine the sequential relationship between the two activities. Similarly, it would be important for researchers to test any such incidents of exposure to simulated gambling (such as those which may be infrequent, such as one or two occurrences in a 12-month period) against other variables or explanations for developing an interest in gambling at the age of 18 years (e.g., social influences and/or personality traits).

### 2.6. Issue 6: Sampling and the Visibility of Adolescent Gambling

Adolescents are generally recognised to be a highly mobile population who spend a great deal of time online. Not only are many parents and teachers unaware of some of the activities with which young people are involved [78,79], but adolescents often communicate in different ways. There is greater use of texting, chat rooms and other forms of social media communication. As a result, there may be a number of challenges associated with gaining insights into adolescent gambling and, in particular, problems associated with gambling. First, it may be very difficult to sample high risk adolescents who never use landlines, who do not answer calls from strangers on their cell-phones, or who use other forms of communication. Second, many of these adolescents may be reluctant to communicate about the extent to their gambling (e.g., if it is illegal or if they have significant financial losses). Finally, it may also be difficult to gain insights into the various factors that influence adolescent gambling because adult researchers may not often know or keep up with how adolescents are interacting with technology (e.g., many of the peer connections may be occurring peer-to-peer in live streams or through social media apps).

## 3. Enhancing Adolescent Gambling Research and Reporting

Although many useful insights have been obtained into the nature of youth gambling, including its prevalence and correlates, there are a number of conceptual and methodological issues that should be examined carefully in future studies. First, it will be important for future studies to include a greater focus on indicators of harm. Instead of inferring harm by examining correlates which may or may not be related to exposure to gambling, researchers should include dedicated questions in relation to gambling-related harm. Questions should capture the different dimensions of harm and the extent to which respondents believe them to be related to gambling as opposed to other influences. A central feature of such analyses would be to include clear measures of expenditure and how this relates to indicators of affordability (e.g., disposable income) as well as other expenditure (i.e., to place gambling in context). Second, in studies that examine the association between different classes of activities and/or the transition to commercial gambling, it is important to examine causality in more detail. To what extent do gamblers migrate to gambling-like games or video-game content as opposed to this content just being appealing to gamblers? What other variables might also account for this behaviour? This will require more nuanced questions and avoiding the assumption that the precedence of one activity (which may be due to legal age restrictions) necessarily involves a valid exposure effect or influence on subsequent gambling. Another important issue relates to the need to be aware of changes in the nature of gambling activities due to the emergence of new activities and technology, including the growth in esports betting [80] or play-to-earn games based on blockchain technology platforms. Finally, we argue that additional studies need to adopt both longitudinal as well as individual level analyses that enable research to gain insights into behaviour within the same individuals. In this way, studies can avoid having to rely on cross-sectional or cohort studies that may conflate group-level stability in behaviour with individual behaviour.

## Data Availability

Not applicable.
